# From wound response to repair – lessons from *C. elegans*

**DOI:** 10.1186/s13619-020-00067-z

**Published:** 2021-02-03

**Authors:** Yicong Ma, Jing Xie, Chandra Sugiarto Wijaya, Suhong Xu

**Affiliations:** 1 The Zhejiang University-University of Edinburgh Institute and Department of Cardiology of the Second Affiliated Hospital, Zhejiang University School of Medicine, Hangzhou, 310058 China; 2 Center for Stem Cell and Regenerative Medicine, School of Basic Medical Sciences, Zhejiang University, Hangzhou, 310058 China

## Abstract

As a result of evolution, the ability to repair wounds allows organisms to combat environment insults. Although the general process of wound healing at the tissue level has been described for decades, the detailed molecular mechanisms regarding the early wound response and rapid wound repair at the cellular level remain little understood. *Caenorhabditis elegans* is a model organism widely used in the field of development, neuroscience, programmed cell death etc. The nematode skin is composed of a large epidermis associated with a transparent extracellular cuticle, which likely has a robust capacity for epidermal repair. Yet, until the last decades, relatively few studies had directly analyzed the wound response and repair process. Here we review recent findings in how *C. elegans* epidermis responds to wounding and initiates early actin-polymerization-based wound closure as well as later membrane repair. We also discussed some remained outstanding questions for future study.

## Background

The efficient healing of a wound is essential for preventing the pathogen invasion, internal tissue loss, and organism survival (Gurtner et al. 2008). Rapid wound healing is indispensable since non-healing wounds such as severe trauma can be fatal, and injury-related mortality takes up 10% of deaths worldwide (Norton and Kobusingye 2013). Delineating the molecular mechanism underlying tissue repair can help people improve their quality of life. Although it is well documented that the main events of wound healing relay on the cooperation of multiple cells, how the tissue and cell immediately detect, respond to, and repair the wounds in vivo remains poorly understood (Enyedi and Niethammer 2015). Thoroughly dissecting the genetic and molecular mechanisms underlying wound repair is essential to develop strategies to intervene or regulate the early cellular response of wounding and to enhance the migration of epidermal cells to heal chronic wounds that cause health and economic burden.

The normal epidermal wound repair process in mammals usually involves three phases at the cellular level (Gurtner et al. 2008). During the early stages of the wound response, platelets and clotting factors gather at the wound site to mediate hemostasis and activate inflammatory cells. The intermediate stage includes the proliferation and migration of epithelial cells and angiogenesis. The later stage consists of remodeling the extracellular matrix (ECM), leading to the recovery of barrier and scar formation (Martin and Nunan 2015). The possibility of infection and secondary damage are positively correlated to the time during which an open wound is exposed to risky extrinsic factors; thus, it is critical for epithelial cells to evolve the ability of early wound detection and rapid response (Enyedi and Niethammer 2015). Previous studies using both invertebrate and vertebrate animal models have revealed that the efflux of damage-associated molecular patterns (DAMPs) from dying cells serve as the initiation factors in the early wound signaling cascade (McDonald et al. 2010; Niethammer 2016; Zhang et al. 2010). However, DAMPs face difficulty in constructing a sophisticated signaling system in the context of epithelial wounds because the environmental or luminal fluid at the wound site may rinse and dilute DAMPs (Niethammer 2016). To establish an extracellular gradient of signal molecules near the wound site, paracrine and transcription-independent signals released by damaged cells at the wound site are strong candidates.

Using anatomically simple and genetically tractable animals as models can illuminate the roles of essential genes and molecules involved in wound response and repair. During past decades, much work has been done by using *C. elegans*, *Drosophila, and* zebrafish, to dissect the logic of wound response and wound repair genetically (Galko and Krasnow 2004; Love et al. 2013; Martin and Lewis 1992; Stanisstreet 1982; Wood et al. 2002; Xu and Chisholm 2011; Yoo et al. 2012; Yoo et al. 2011). Besides, many transcriptional-independent signals were demonstrated as immediate responses to wounding (Cordeiro and Jacinto 2013). For example, researchers found that wounding induces Ca^2+^-dependent dual oxidase (DUOX) production of H_2_O_2_ gradient and mediate the rapid recruitment of leukocytes (Niethammer et al. 2009). Subsequent studies find that H_2_O_2_ responding to wounding plays a conserved role in multiple organisms required for tissue repair and regeneration (Love et al. 2013; Razzell et al. 2013; Suzuki and Mittler 2012; Yoo et al. 2012).

*C. elegans* is an emerging model organism in the context of regenerative medicine due to its access to live imaging and diverse genetic techniques (Xu et al. 2012). With the help of live imaging, it is relatively efficient to investigate the wound detection, response, and repair processes (Xu and Chisholm 2014b) (Table 1). Wounding the epidermis triggers at least two parallel responses, including innate immune response and rapid wound repair response (Chisholm 2015; Chisholm and Xu 2012). The innate immune response involves the upregulation of a suite of antimicrobial peptides (AMPs) (Pujol et al. 2008). Wound repair response involves a Ca^2+^ mediated rearrangement of the actin cytoskeleton (Xu and Chisholm 2011). Interestingly, mitochondria can actively respond to wounding and protect from damage to promote actin-polymerization-based wound closure (Fu et al. 2020; Xu and Chisholm 2014a). These processes appear to be initiated independently, yet, their coordinated activity ensures the animal to survive otherwise fatal epidermal wounds. In this article, we review the recent works focusing on the wound response and repair process in *C. elegans* epidermis.

**Table 1:** Time-courses of the wound response and repair in *C. elegans* epidermis

Phase	Key Biological Processes	Time
Response phase (Transcriptional- independent)	Ca^2+^ influx mitochondrial Ca^2+^ uptake Mitochondrial fragmentation mtROS production Actin polymerization	Milliseconds –seconds Seconds Seconds Seconds - minutes Minutes
Repair phase (Transcriptional activation)	Cytochrome P450 upregulations SYX-2 and EFF-1 induction AMPs induction SYX-2 recruitment EFF-1 recruitment	30 min to hours 30 min to hours Hours 30 min to hours Hours
Remodeling phase	scar formation	> 6 h

The events listed above are highly interconnected with spatial-temporal overlaps

## *C. elegans* epidermis as a model to study wound response and repair

**Fig. 1:**
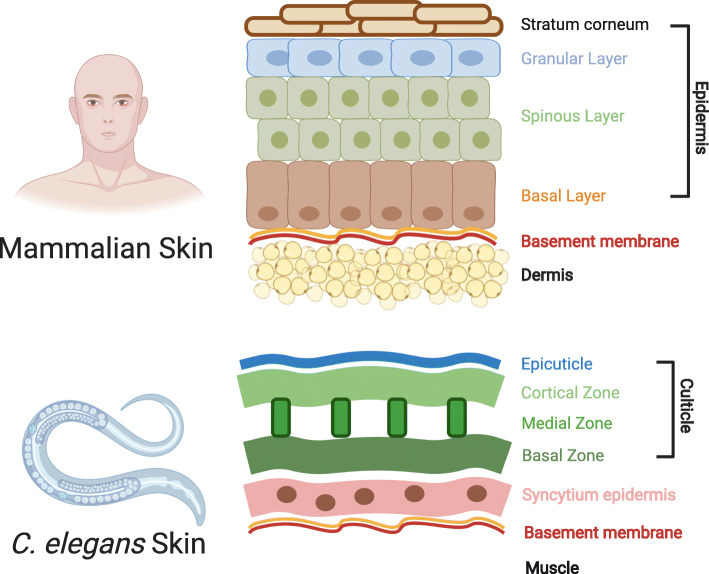
The difference between mammalian and *C. elegans* skin. *C. elegans* attains a simple but tenacious body structure through evolution. During the embryonic stage, a group of epidermal cells undergo a series of fusions and to develop a large multinucleated syncytium hyp7. The syncytium will secrete the cuticle, an overlaid sheath of an extracellular matrix consisting of collagen, lipids, and glycoproteins. As a result, there are two layers within *C. elegans* skin: epidermis and cuticle. When the worm gets fully matured, the skin gets postmitotic and can only grow through polyploidization. On the other hand, the mammalian skin is a stratified epithelium made of keratinocytes in each layer. The basal layer contains stem cells that can proliferate and push daughter cells to migrate toward the surface. Due to the lack of oxygen and water around the outer layers, keratinocytes will die and cornify at the surface. These dead cells can compose the stratum corneum, which is the primary permeability barrier of the skin

*C. elegans* skin is composed of a simple epidermal epithelium syncytium and an external cuticle (Chisholm and Hsiao 2012) (Fig. 1). Although the skin structure of *C. elegans* is different from that of the mammals, it exhibits several similarities in wound response and repair, including activation of the innate immune system and remodeling of the permeability barrier (Xu et al. 2012). Compared to mammals, the epidermal syncytium of *C. elegans* is closer to a single-cell wound model, lacking cell division and angiogenesis, which are essential steps in mammalian skin regeneration (Eming et al. 2014). However, as a differentiated barrier epithelium model, the simple structure and transparent layers provide an ideal platform to trace molecular changes both in a short time and a large scale. The wound healing process after epidermal injury allows organisms to regain the epithelial integrity. By targeting this process, potentially novel therapeutic strategies could be developed to treat wounds in various pathophysiological settings.

## Epidermal wounding induces transcriptional-dependent innate immune responses

The first wound response to be elucidated in the worm is the transcriptional regulated innate immune response in the epidermis. The nematode skin is under hydrostatic pressure; therefore, puncture damage can be fatal if not rapidly healed (Kurz and Ewbank 2000). Such damages may be common in nature, where nematodes frequently encounter damaging substrates and cuticle-puncturing pathogens (Vidal-Diez de Ulzurrun and Hsueh 2018). Analysis of the epidermal innate immune response to damage began with pioneering studies of skin-penetrating pathogens such as *Drechmeria coniospora*, which generate invasion spores that stick to the cuticle extend hyphae through the underneath epidermis to kill the animal eventually (Jansson 1994). Fungal infection specifically induces epidermal expression of a large set of antimicrobial peptides (AMPs), including a MAPK cascade induced the neuropeptide-like (*nlp*) genes (Couillault et al. 2004), and a TGF-β cascade induced caenacin (*cnc*) genes (Zugasti and Ewbank 2009).

The process of skin penetration by fungal hyphae is reminiscent of epidermal damage, leading to the question of whether innate immune responses to infection are specific to the pathogen or are more general responses to the damage. Using needles or lasers to wound the skin, Pujol et al. showed that physical damage was sufficient to induce some of the epidermal AMPs activated by infection, through the same signaling cascade involved in AMP induction after infection (Pujol et al. 2008). The Toll-Interleukin-1 Receptor (TIR) domain adaptor protein TIR-1 (orthologous of mammalian SARM) could trigger the p38 MAPK pathway, and TIR-1 itself is activated by protein kinase C (PKC) TPA-1(Couillault et al. 2004; Ziegler et al. 2009). Wounding also induce the TGF-β cascade dependent expression of CNCs (Zugasti and Ewbank 2009), but how TGF-β signaling responds to wounding is not yet clear. It is worth to note that the epidermis can also recognize the damage through hemidesmosomes associated with a STAT-like protein, whose disruption led to the detachment of STA-2 molecules from hemidesmosomes and initiation of the AMPs induction (Zhang et al. 2015), indicating that diverse strategies might be used as a response to epidermal damage that triggers the active innate immunity to protect from infection.

Nonetheless, there are some differences between the innate immune response to pathogen infections and skin wounding. First, some genetic mutations, such as the NIPI-3 mutant, only block the infection-specific branch of the signaling pathway but not wounding (Pujol et al. 2008). Second, two of six nematode phospholipase C genes, PLC-3 and EGL-8, act upstream of PKC TPA-1, and the response to physical damages is primarily influenced by PLC-3 but not EGL-8 (Ziegler et al. 2009). Despite the diversity of signaling, the upstream of phospholipase C, Gα, and Gβ protein genes GPA-12 and RACK-1, could be induced by both fungal hyphae and physical injury (Ziegler et al. 2009; Zugasti et al. 2014). G protein signaling in the innate immune response to wounding indicates that one or more GPCRs might be able to sense tissue damage, which will be a promising avenue for future investigation.

Interestingly, the wound-induced innate immune response is negatively regulated by a death-associated protein kinase, DAPK-1 (Tong et al. 2009). A point mutation in *dapk-1(ju4)* displays constitutively elevated levels of epidermal AMPs, and genetic interaction studies indicate that DAPK-1 acts upstream of p38 MAPK pathways. The gain-of-function of GPA-12 also displays a constitutively elevated expression of NLPs (Ziegler et al. 2009). This constitutively innate immune response defends against opportunistic infection at wounds, since p38 MAPK mutants display reduced survival after epidermal wounding (Tong et al. 2009; Xu and Chisholm 2011). However, the p38 MAPK cascade was not shown to be required for other wound healing processes, such as wound closure and scar formation (Pujol et al. 2008; Xu and Chisholm 2011). How DAPK-1 regulates p38 MAPK cascade activity remains to be investigated.

## Epidermal wounding triggers direct actin polymerization that drives wound closure

Diverse early wound signaling cascades share a common goal that the epidermal damage should be healed and recovered immediately. The physical breach on the epidermis of the nematode will be patched with the help of nearby dynamic cytoskeleton and membrane vesicles beneath it. Recent findings indicate that wounding triggers a rapid actin polymerization, which forms into actin rings surrounding the wound site to close the wound (Xu and Chisholm 2011). Importantly, efficiently closure of these actin rings is required for the post-wounding survival of the animal. The actin cytoskeletal dynamics after the injury have also been discovered in other animal models. In the *Drosophila* embryo, the wound site was closed by actomyosin cables in a “purse-string” manner (Martin and Lewis 1992; Wood et al. 2002), whereas in *Xenopus* oocyte, the closure of the injury requires both actin cable formation and Ca^2+^ activation (Benink and Bement 2005; Clark et al. 2009).

**Fig. 2:**
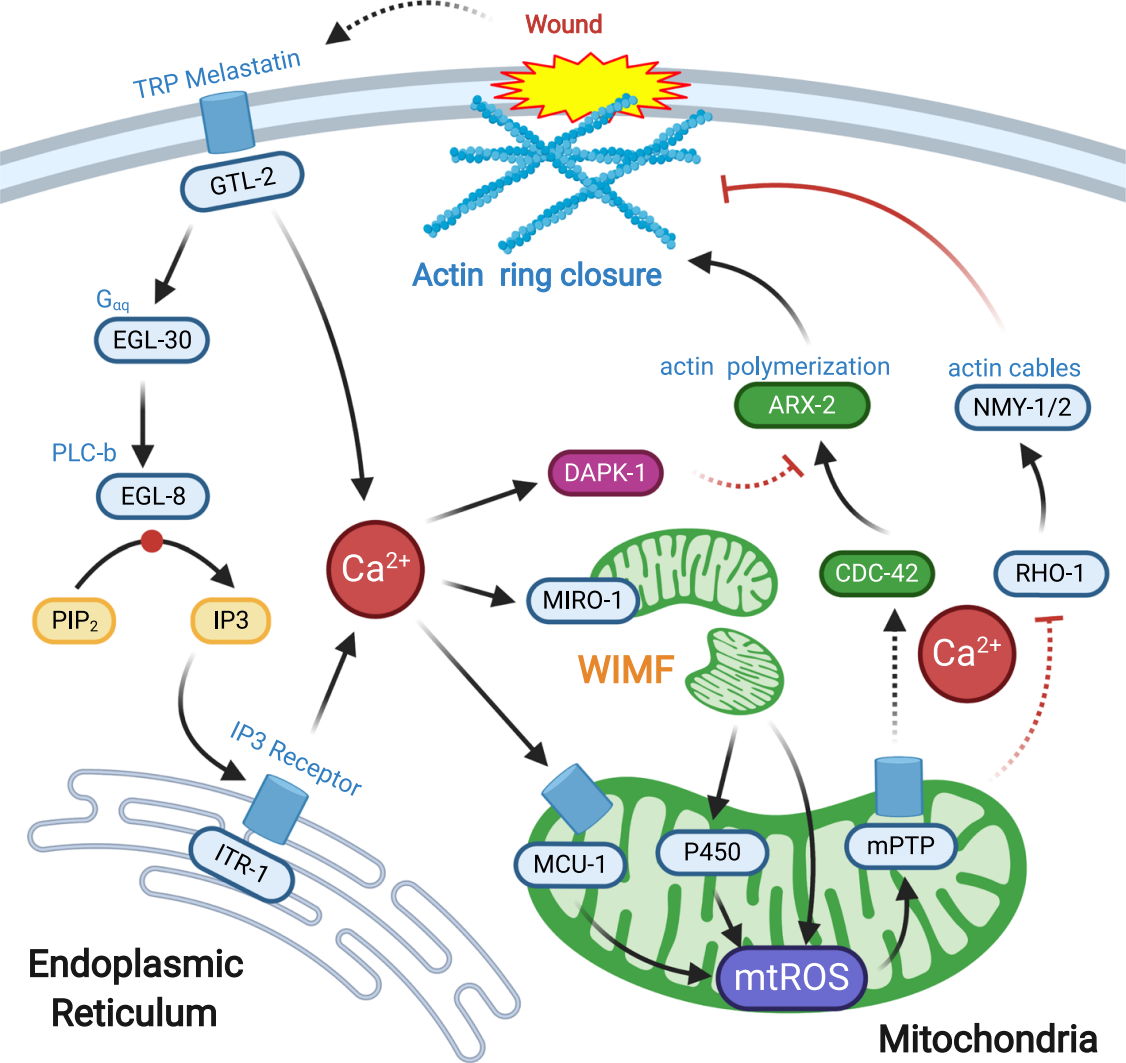
Wounding induces Ca^2+^ and mitochondrial responses that promote actin-polymerization to repair the wound. Wounding can trigger an instant rise in the epidermal cytosolic Ca^2+^ level. TRPM channel GTL-2 in the plasma membrane and IP3 receptor ITR-1 located at the endoplasmic reticulum contributes to the initial of Ca^2+^ activation. Through the mitochondrial Ca^2+^ uniporter MCU-1, cytosolic Ca^2+^ enters into the mitochondria matrix and triggers the production of mtROS. Besides, Ca^2+^ also regulates wound-induced mitochondrial fragmentation (WIMF) through the outer mitochondrial membrane protein MIRO-1 to enhance the mtROS signals. The epidermal wound is essentially closed by direct actin polymerization, which is dependent on Ca^2+^ activation. mtROS regulates the local activation of small GTPases RHO-1 to promote actin polymerization based wound closure

Importantly, wounding induced actin cytoskeleton is not an actomyosin cable but rather a CDC-42 small GTPase and Arp2/3(ARX-2 in worms) dependent direct actin polymerization (Xu and Chisholm 2011) (Fig. 2). In contrast to actomyosin cables in *Drosophila* embryonic and larvae wounding (Galko and Krasnow 2004; Martin and Lewis 1992), the nematode actin ring formation is negatively regulated by RHO-1 and non-muscle myosin (NMY), including myosin heavy chain NMY-1/2 and myosin light chain MLC-4 (Xu and Chisholm 2011). RHO-1 and CDC-42 might directly antagonize, as described in *Xenopus* oocyte wounding (Vaughan et al. 2011). Alternatively, the enhanced closure seen after inhibition of RHO-1 or NMY-1/2 might be an indirect consequence of the reduction in actin cable formation at the wound site. Thus, in this sense, epidermal wound closure in *C. elegans* might resemble the repair mechanisms in other adult epithelia, in which the repair is mostly driven by filopodial protrusive activity at the leading edge (Sonnemann and Bement 2011). It would be interesting to determine whether and how RHO-1 and CDC-42 respond to wounding for the locally simultaneously activation in the future.

Like DAPK-1 negatively regulates the wounding-induced innate immunity, a point mutation of *dapk-1(ju4)* also results in a faster actin-ring based wound closure and hypertrophic cuticle growth (similar to hypertrophic scar formation) at the head region (Tong et al. 2009; Xu and Chisholm 2011). The similarity in the effects of DAPK-1 and non-muscle myosin on wound closure can be accounted for since the myosin light chain is a known target of Dapk1 in mammalian cell (Bialik et al. 2004). The inhibitory role of DAPK-1 in the wound closure, together with previous evidence that DAPK-1 inhibits the response of innate immunity subsequent to damage. In light of that, DAPK-1 can act as a negative coordinate regulator for both innate immunity and wound repair pathways (Tong et al. 2009; Xu and Chisholm 2011). Recently, a forward genetic screen revealed that a point mutation on the gene *ptrn-1*, which encodes the microtubule minus-end binding protein Patronin (Nezha homology in mammals), could completely suppress either epidermal or innate immunity phenotype in the *dapk-1* mutant (Chuang et al. 2016), suggesting an unexpected interdependence of DAPK-1 and the microtubule cytoskeleton maintenance of epidermal wound repair and integrity. However, how microtubule dynamics regulate epidermal wound closure remains little understood.

## Epidermal wounding induces immediate transcriptional-independent Ca^2+^ elevation in vivo

How does the epidermal cell sense the damage and initiate rapid innate immune responses as well as actin polymerization-based wound closure? Generally, Ca^2+^ takes part in various cellular functions, and its critical role in the repair process has been revealed at the cellular level (Lansdown 2002; Stanisstreet 1982). Thanks to the application of genetically encoded Ca^2+^ sensor GCaMP3, a fusion protein expressed by the transgenic worm, it becomes possible to trace the spread of Ca^2+^ inside the epidermis of *C. elegans* (Xu and Chisholm 2011) (Fig. 2). Both laser and needle wounding triggers immediate elevation of Ca^2+^ that can persist for at least 1 h. Candidate RNAi screening result found that the knockdown of membrane-bounded *gtl-2* (TRPM channel) or *itr-1* (IP3R on the Endoplasmic Reticulum) significantly reduced Ca^2+^ elevation after wounding, suggesting that both extracellular and intracellular store contributes to the wounding-induced Ca^2+^ elevation. Further genetic identification finds that a Gαq EGL-30 and its effector PLC-β EGL-8 are required for epidermal Ca^2+^ signaling, acting through the ITR-1 (Xu and Chisholm 2011). Interestingly, a *Drosophila* injury response requires the function of the TRP channel TRPM (Antunes et al. 2013) and Brv1 (Turner et al. 2018). In zebrafish, TRPV1 functions in keratinocyte migration, mechanistically relevant to wound healing (Graham et al. 2013). Whereas the exact role of TRP channels in epidermal Ca^2+^ homeostasis is likely to evolve differently between species, these findings suggested that TRP channels are both conserved and plays a crucial role in wound response and repair.

Wounding-induced Ca^2+^ signaling is not required for innate immune response as Ca^2+^ chelator BAPTA-AM did not affect AMP induction, but significantly reduced the survival rate by inhibiting actin polymerization (Xu and Chisholm 2011). Actin polymerization is also blocked in the *gtl-2* mutant, the defects of which can be partially rescued by incubation in buffers with high external Ca^2+^concentration (Xu and Chisholm 2011). In *Xenopus* oocyte, the wounding of a single cell activates both small GTPase Cdc42 and Rho, via a Ca^2+^ dependent signal (Benink and Bement 2005; Clark et al. 2009). It is reasonable to postulate that epidermal wounding induces the elevation of Ca^2+^, which subsequently triggers the local activation of these small GTPases. If so, an important goal in the future will be to define molecules responsible for GTPase activation in response to wounding and to dissect how these themselves are regulated in the epidermis.

## Wounding induces mitochondrial Ca^2+^ uptake dependent mitochondrial ROS production in the epidermis

Mitochondria are the energy hub of the cell that responds to metabolic signals and produce ATP to support cellular homeostasis. Unexpectedly, we observed that for the first time, epidermal wounding could dramatically trigger the activation of mitochondrial ROS (mtROS) superoxide sensor mito::cpYFP flash around the wound site (Xu and Chisholm 2014a), suggesting an elevated level of mtROS produced in the mitochondria after wounding. Increased production of mtROS as a consequence of MCU-1 dependent mitochondrial Ca^2+^ uptake (Xu and Chisholm 2014a). Based on the time courses of mitochondrial Ca^2+^ uptake and mitoflashes after wounding, the initial Ca^2+^ uptake by mitochondria may trigger the mtROS production through the opening of the mitochondrial permeability transition pore (mPTP), whose molecular identity remains elusive yet. mtROS levels were also shown to be elevated in fibroblasts wounding (Janda et al. 2016), the injured skeletal muscle (Horn et al. 2017), and also in *Drosophila* dorsal closure (Muliyil and Narasimha 2014), a developmental process analogous to aspects of wound healing. These findings demonstrate that mtROS may play a conserved role in regulating wounds in a barrier epithelium.

Further studies have shown that mtROS play protective roles in skin wound repair in vivo (Fig. 2). The mtROS burst can locally inhibit a small GTPase RHO-1 and promotes direct actin polymerization to close the wound hole (Xu and Chisholm 2014a). Conversely, inhibition of mtROS by antioxidant treatment blocks wound closure. The elevated level of mtROS causes local inhibition of RHO-1 activity by targeting on a redox-sensitive motif, enhancing the actin-based wound closure. Recently, Ca^2+^ triggered mtROS production facilitating wound closure has been shown in injured skeletal muscle cell repair in mice, and RhoA is activated to promote F-actin accumulation for wound healing (Horn et al. 2017). In mammals, tissue injury-induced mitochondrial oxidative phosphorylation regulates the repair of multiple tissues, including the epidermis (Cano Sanchez et al. 2018; Janda et al. 2016). Thus, mitochondria play diverse roles in tissue repair after damage, and manipulation of mtROS may be of interest in therapies for accelerating tissue repair.

Although mtROS shows its positive influence on promoting actin polymerization and wound closure in *C. elegans*, excessive ROS accumulation in humans is commonly known as oxidative stress, which causes the impaired wound healing in patients with diabetes or treated with chemo- or radiotherapy (Schafer and Werner 2008). For example, by measuring the concentration of 8-isoprostanes in fluid from chronic venous ulcers, researchers detect the peroxidation of fatty acids with a high ROS level (Yeoh-Ellerton and Stacey 2003). Due to the substantial oxidizing property of ROS, both *C. elegans* epidermis and human skin may suffer from oxidative stress, which interferes with the normal repair process. Thus, the balance of generation and detoxification of ROS inside the cell should be considered for wound repair. The mechanism preventing excess mtROS production in *C. elegans* is worth to be investigated, and considering the short half-life of ROS, reactive oxygen is often converted to more-stable molecules such as H_2_O_2_ (Xu and Chisholm 2014a), and the regulation of H_2_O_2_ production may play an important role in controlling the level of mtROS.

## Wounding induces mitochondrial fragmentation to promote wound repair

It is well known that mitochondria form a highly dynamic tubular network within cells, reflecting a balance of fusion and fission events linked to the ATP production and oxidative metabolic requirements of the cell survival. Our recent study found that wounding also triggers rapid and reversible mitochondrial fragmentation, a process refers to as wounding induced mitochondrial fragmentation (WIMF) (Fu et al. 2020). Moreover, U2OS cell scratch wounding and zebrafish tailfin wounding also show similar mitochondrial fragmentation phenotype (Fu et al. 2020), suggesting that WIMF may be a general wound response mechanism. Importantly, loss of function in two genes, *fzo-1*(Mfn1/2 homology) and *eat-3*(Opa1 homology), whose activities are required for mitochondrial fusion (Hoppins 2014), leads to chronic mitochondrial fragmentation and faster-wound closure (Fu et al. 2020). Consistent with this finding, mitochondrial fragmentation has also been found to promote cellular repair (Horn et al. 2020) and *Drosophila* embryonic wound healing (Ponte et al. 2020), suggesting WIMF is not only a rapid wound response but also plays a conserved role in regulating wound repair.

How wounding triggers mitochondrial fragmentation? An outer mitochondrial membrane protein RNAi screen was conducted to identify the potential molecule responsible for sensing the wounding signal. The result showed that WIMF does not depend on the master fission regulator DRP-1 but instead requires the Ca^2+^-sensitive mitochondrial Rho GTPase MIRO-1 and cytosolic Ca^2+^ (Fig. 2). Interestingly, Nemani et al. also reported recently that elevated cytosolic Ca^2+^ induces a mitochondrial shape transition in HeLa and MEF cells are dependent on MIRO1 but not DRP1 (Nemani et al. 2018), suggesting Ca^2+^-MIRO-1 plays a vital role in regulating mitochondrial morphology under diverse stresses and wounding. Moreover, a recent study report that plasma membrane injury in MEFs induces Drp1-mediated mitochondrial fragmentation, which enables localized signaling required for cell repair (Horn et al. 2020). It would be fascinating to investigate whether and how wounding induced mitochondrial fragmentation through different downstream targets in diverse models. Possibly the amplitude or local nature of the wound-induced Ca^2+^ transient is sufficient to trigger a more rapid mitochondrial fragmentation response via diverse downstream molecules. How Ca^2+^ regulates MIRO-1 or Drp1 through their local activity cause mitochondrial fragmentation remains to be determined.

Ca^2+^ mediates diverse biological processes, including synapse activation, neuronal activity, fertilization, etc. These are unrelated to tissue injury, raising the question of how cells distinguish between wound-induced and physiological Ca^2+^ transients. Previously, our study has shown that wounding triggers mtROS production, mediated by MCU-dependent mitochondrial Ca^2+^ uptake, and that mtROS can promote wound healing (Xu and Chisholm 2014a). We recently found that MIRO-1 is another key downstream target of Ca^2+^, which is independent of MCU-1 dependent Ca^2+^ uptake into mitochondria (Fu et al. 2020). Thus, wound-induced Ca^2+^ signals may act via multiple effectors to generate a protective mtROS cascade in regulating tissue repair. The other endogenous targets of wounding-induce Ca^2+^ influx remains to be discovered.

## Mitochondrial oxidative and cytochrome P450 signal in wound response and wound repair

Genetic and transcriptomic analyses have shown that enhanced mitochondrial fragmentation accelerates wound closure via the upregulation of mtROS and Cytochrome P450 (CYPs). Our study found that mitochondrial fragmentation can trigger mtROS production and expression of oxidative signaling genes like CYP-13A8 (cytochrome P450 in humans), which in turn maintains a high level of reactive oxygen species (ROS), resulting in the improvement on wound closure (Fu et al. 2020) (Fig. 2). Moreover, overexpression of *cyp-13A8* in nematode epidermis enhances mtROS and promote wound closure. CYPs have been shown to respond to wounding and accelerate wound epithelization in diabetic mice (Zhao et al. 2017), hairless mouse ear (Sander et al. 2011), and even in plants (Noordermeer et al. 2001). This study, consistent with others, suggests that CYPs may be vital in mediating oxidative signals that promote damage repair, although it is so far unclear how CYPs respond and triggers the oxidative signal to regulate later wound repair. By uncovering a link between mitochondria, CYPs signaling, and wound repair, studies in nematode may open the door to novel therapeutic interventions based on mitochondrial signals.

## Wounding induced transcriptional activation of membrane fusion genes that regulate epidermal membrane repair

A fundamental step of wound repair in a single cell is rebuilding the damaged plasma membrane to restore the cellular homeostasis and function (Sonnemann and Bement 2011). The epidermis of *C. elegans* consists of a syncytium hyp7 that contains 139 nuclei and is the largest somatic cell (equivalent to a giant cell) (Chisholm and Hsiao 2012), the wound repair process of nematode skin is closely related to the membrane repair. It is known that plasma membrane repair requires coordinated activation of several cytosolic pathways, as well as rearrangement from sequential recruitment of different vesicle components to the wound site to restore internal cellular homeostasis and prevent cell death. However, how the hyp7 repair its damaged membrane in the living animal was not known.

**Fig. 3:**
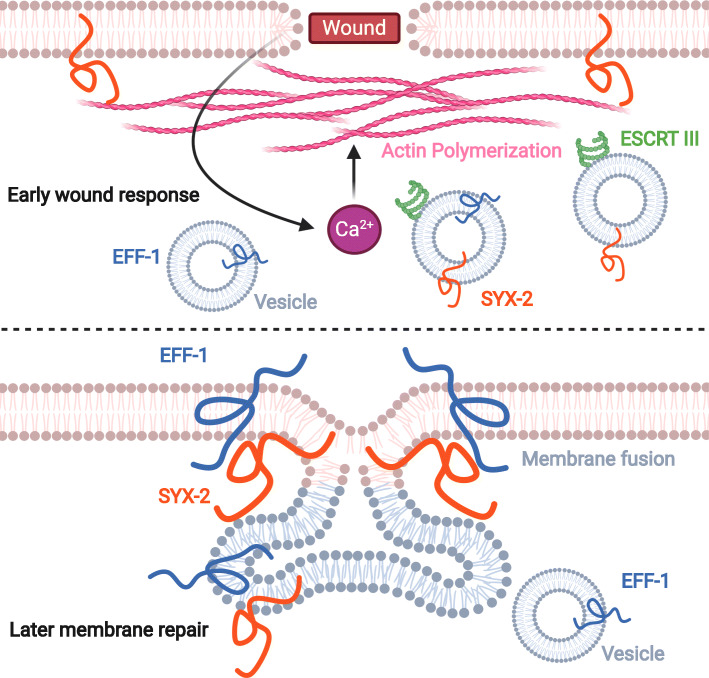
ESCRT III, SYX-2, and EFF-1 sequential recruitment to regulate membrane repair. *C. elegans* epidermal membrane repair requires the sequential recruitment of ESCRTIII, SYX-2, and EFF-1 to the wound site. Through exocytosis or endocytosis, pre-existing intracellular vesicles can patch the open wound to carry out membrane repair. As a result of the early wound response, both actin polymerization and Ca^2+^-regulated ESCRT III signals are required for SYX-2 and EFF-1 recruitment to the wound site

Recently we applied single worm RNA sequencing to investigate the transcriptional regulation after epidermal wounding and found that the epithelial-fusion failure (*eff-1*) gene was highly upregulated (Meng et al. 2020) (Fig. 3). Moreover, EFF-1 protein can be rapidly recruited to the wound site and is required for membrane repair and animal survival. EFF-1 encodes a transmembrane protein with structural homology to viral class II fusion proteins, which is essential for epidermal cell fusion in development (Mohler et al. 2002; Perez-Vargas et al. 2014; Shemer et al. 2004). Interestingly, EFF-1 not only functions as a cell-cell fusion protein (Gattegno et al. 2007; Mohler et al. 2002; Rasmussen et al. 2008; Shemer et al. 2004) but also acts in repairing severed axons (Basu et al. 2017; Ghosh-Roy et al. 2010; Neumann et al. 2015), maintenance of dendritic arborization (Oren-Suissa et al. 2010; Zhu et al. 2017), and sealing of phagosomes (Ghose et al. 2018), suggesting that EFF-1 might play conserved functions in diverse plasma membrane repair after cellular damage. In the damaged epidermis, the accumulation of EFF-1 at the wounded membrane is dependent on the early Ca^2+^ regulated actin polymerization and the SNARE protein Syntaxin2 (SYX-2). SYX-2 interacts with the C-terminal of EFF-1 to promote EFF-1 localization, an event that may facilitate both intracellular and extracellular membrane repair (Meng et al. 2020) (Fig. 3). It would be interesting to investigate whether and how SYX-2 and EFF-1 repair machinery functions in other membrane repair processes.

Multiple evidence has shown that Ca^2+^ regulated exocytosis of pre-existing intracellular vesicles into membrane patches, exocytosis of lysosomes, ESCRT machinery, and membrane lesion removal by endocytosis are all involved in the repair of membrane wounds in a single cell in vitro (Andrews and Corrotte 2018). Our study found that wounding can also induce rapid recruitment of VPS-32.1 (CHAM4B homology), which is a Ca^2+^-regulated ESCRT III component, and VPS-4 (VPS4 homology) (Meng et al. 2020), suggesting that ESCRT signal plays a conserved role in regulating membrane repair. More strikingly, epidermal specific RNAi knockdown ESCRT components significantly inhibited SYX-2 and EFF-1 recruitment, demonstrating that the sequential recruitment of endoplasmic membrane-localized SYX-2 and exoplasmic membrane fusion gene EFF-1 were dependent on ESCRT III signal, reflecting a potential link between membrane curvature and wound repair. However, how Ca^2+^ dependent ESCRT III regulates localization and recruitment of SYX-2 and EFF-1 is not yet entirely clear, potentially due to the essential role of critical vesicles that contain SYX-2 and EFF-1. Moreover, how the two fusion protein, SYX-2 and EFF-1, coordinated with each to repair the damaged membrane is not clear. Detailed characterization of the biochemical and biophysical mechanism of SYX-2 and EFF-1 machinery will be crucial for understanding how wounds heal efficiently and effectively in the future.

## Conclusion and remaining questions

It is still early days for *C. elegans* epidermal wound healing studies. However, it is clear that the genetic tractability of *C. elegans*, combined with the various live imaging opportunities available, will become an even more potent contributor to our understanding of fundamental mechanisms that underpin wound detection, response, and membrane repair. Many questions remain to be answered form initial wound response to the end of the membrane repair. For example, how early wound signals trigger sequential and spatial transcriptional activation are not yet known, nor is the precise mechanism leading to the perfect remodeling of the epidermis. Current studies have established worm epidermis as a new system for membrane repair in the living animal; however, the underlying molecular mechanism for the membrane repair process in either adult or aged animals is poorly understood. Unlike zebrafish or Drosophila larvae wound healing systems, which show robust repair or regeneration abilities, the *C. elegans* skin repair itself often results in cuticle scarring (Pujol et al. 2008; Tong et al. 2009), yet it is not clear what the scar is and how is formed after the damage.

The powerful genetic screens will undoubtedly reveal novel genes and signaling pathways involved in wound response and repair in vivo that can be further tested in mammals; indeed, the results of nematode studies are already influencing how we approach questions about wound healing in vertebrates. With this proviso, *C. elegans* now seems to be an important model for studying wound response, repair, and remodeling, beautifully complementing research carried out in other species, including flies, zebrafish, Xenopus, and mice.
